# Exo70 is transcriptionally up-regulated by hepatic nuclear factor 4α and contributes to cell cycle control in hepatoma cells

**DOI:** 10.18632/oncotarget.7133

**Published:** 2016-02-02

**Authors:** Yujie Zhao, Jihuan Hou, Panying Mi, Liyuan Mao, Liang Xu, Youyu Zhang, Li Xiao, Hanwei Cao, Wenqing Zhang, Bing Zhang, Gang Song, Tianhui Hu, Yan-yan Zhan

**Affiliations:** ^1^ Cancer Research Center, Xiamen University Medical College, Xiamen 361102, Fujian Province, PR China; ^2^ Department of Oncology, Zhongshan Hospital Affiliated to Xiamen University, Xiamen 361004, Fujian Province, PR China; ^3^ Department of Basic Medicine, Xiamen University Medical College, Xiamen 361102, Fujian Province, PR China

**Keywords:** Exo70, HNF4α, transcription, cell cycle regulation, hepatoma

## Abstract

Exo70, a member of the exocyst complex, is involved in cell exocytosis, migration, invasion and autophagy. However, the expression regulation and function of Exo70 in hepatocellular carcinoma are still poorly understood. In this study, we found Exo70 expression in human hepatoma cells was greatly reduced after knocking down hepatic nuclear factor 4α (HNF4α), the most important and abundant transcription factor in liver. This regulation occurred at the transcriptional level but not post-translational level. HNF4α transactivated Exo70 promoter through directly binding to the HNF4α-response element in this promoter. Cell cycle analysis further revealed that down-regulation of HNF4α and Exo70 was essential to berberine-stimulated G2/M cell cycle arrest in hepatoma cells. Moreover, knocking down either Exo70 or HNF4α induced G2/M phase arrest of hepatoma cells. Exo70 acted downstream of HNF4α to stimulate G2/M transition via increasing Cdc2 expression. Together, our results identify Exo70 as a novel transcriptional target of HNF4α to promote cell cycle progression in hepatoma, thus provide a basis for the development of therapeutic strategies for hepatocellular carcinoma.

## INTRODUCTION

Exocyst, a heterooctameric complex composed of Sec3, Sec5, Sec6, Sec8, Sec10, Sec15, Exo70 and Exo84, is essential for exocytosis via tethering secretory vesicles to specific domain of the plasma membrane [1–4]. Recent studies implicated the pivotal roles of its subunit Exo70 in multiple biological processes. Overexpression of Exo70 dominant-negative mutant blocks insulin-stimulated glucose uptake through inhibiting GLUT4 vesicle targeting to the plasma membrane in adipocytes [5–8]. Knocking down Exo70 in human breast cancer cell suppresses cell invasion via attenuating invadopodia formation and matrix metalloproteinases secretion [9, 10]. Exo70 is also necessary for amino acid starvation induced autophagy in human cervical carcinoma cells [11]. However, little is known about the expression, regulation and function of Exo70 in hepatocellular carcinoma (HCC).

Hepatocyte nuclear factor 4 alpha (HNF4α), a member of the nuclear receptor superfamily, is the most abundant and important transcription factor in the liver [12–14]. HNF4α exerts its biological functions primarily through binding as a homodimer to the HNF4α-response element (HRE) in the promoter of its downstream target genes and regulating their expression [15, 16], including apolipo-protein A-I (ApoA-I) [17], lipid transporter ABCA1 [18], complement C3 [19], murine pyruvate carboxylase (PC) [20], EMT-related genes [21], and over 60 other genes critical for metabolism, nutrient transport and the development of human diseases including diabetes, haemophilia and hepatitis [22, 23]. Therefore, the identification of new downstream target genes may help to discover new biological functions of HNF4α and understand how it works.

In the present study, we provide evidences that HNF4α transcriptionally regulates Exo70 gene expression in human hepatoma cells through binding to the HNF4α-response element (HRE) within the *Exo70* promoter, which contributes to the G2/M cell cycle transition.

## RESULTS

### HNF4α transcriptionally increases the expression of Exo70 in hepatoma cells

**Figure 1:**
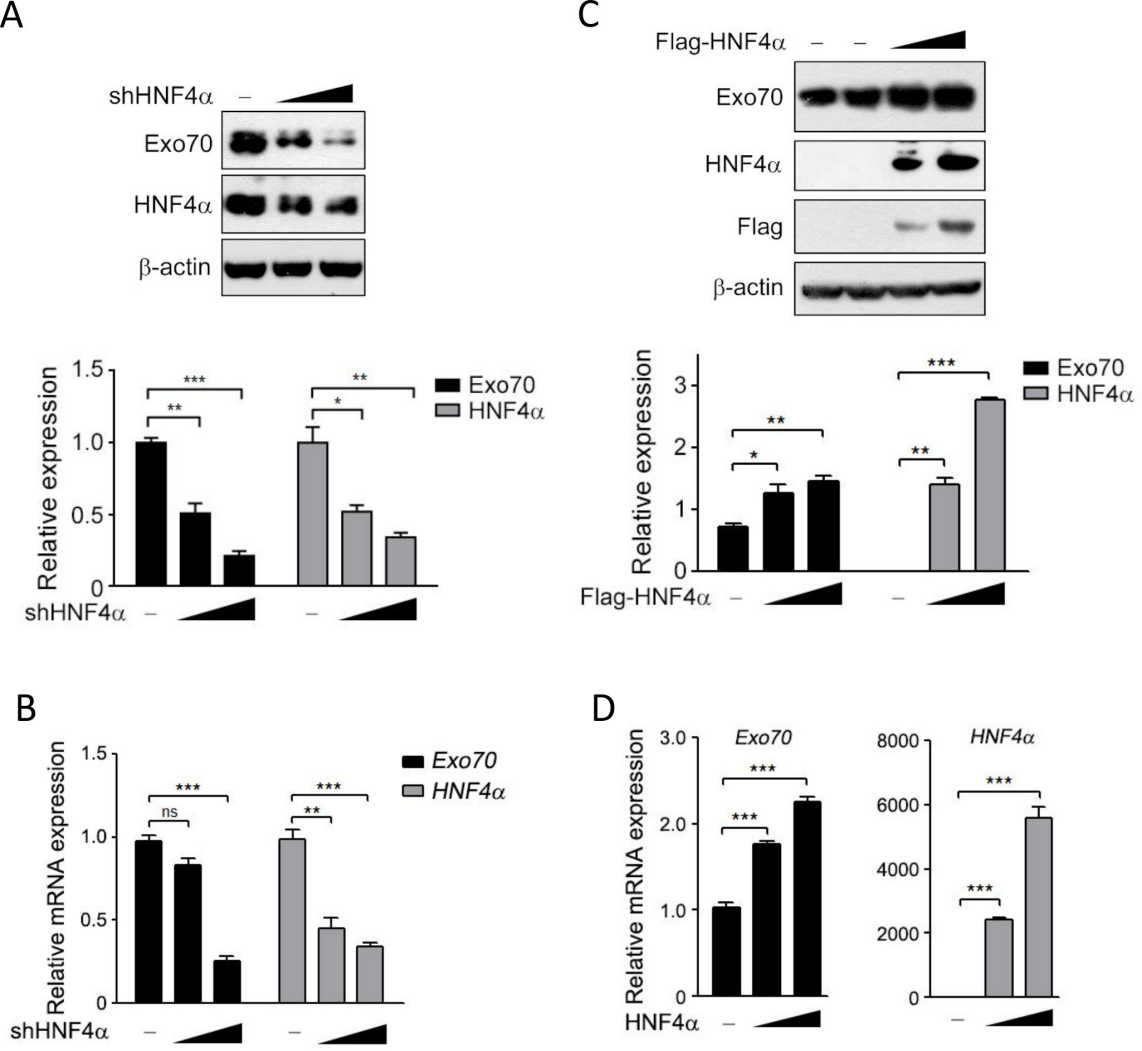
HNF4α regulates the expression of Exo70 protein (**A**–**B**) Knocking down HNF4α decreased the protein and mRNA expression of Exo70. Hep G2 cells were transiently transfected with pLL3.7 vector or pLL3.7-shHNF4α plasmid. Cells were harvested 48 h after transfection and the whole cell lysate and the total RNA were prepared then subjected to Western blot (A) and real time PCR (B) analysis. (**C**–**D**) Ectopically expressed HNF4α upregulated the protein and mRNA expression level of Exo70 in cholangiocarcinoma cell line QBC939. Cells were transfected with pCMV10 vector or Flag-HNF4α plasmid, and harvested 48 h after transfection and subjected to Western blot (C) and real time PCR (D) analysis. Protein expression of Exo70, HNF4α and β-actin (used as loading control) were detected and quantified by densitometry; bar graph (A, C, down panel) showed the ratios of Exo70 or HNF4α to β-actin, and the basal level in the vector group was normalized to 1. GAPDH was used as normalization control for real time PCR. Data were presented as the means ± s.e.m. from three independent experiments. Differences between two groups as indicated in the graph were assessed by a two tailed unpaired Student's *t*-test, *p < 0.05, **p < 0.01, ***p < 0.001 versus control.

To investigate whether hepatic Exo70 expression was regulated by HNF4α, the most important and abundant transcription factor in liver, we knocked down endogenous HNF4α in human hepatic cancer cell line Hep G2, and a dramatic decrease in the protein and mRNA expression level of Exo70 was observed (Figure 1A–1B). Conversely, ectopically expressing HNF4α in human cholangiocarcinoma QBC939 cells, which have extremely low endogenous HNF4α but derived from the same embryonic origin with liver, resulted in a significant increase in the protein and mRNA expression levels of Exo70 (Figure 1C–1D). These results indicated that Exo70 expression was elevated by HNF4α in hepatoma cells.

**Figure 2:**
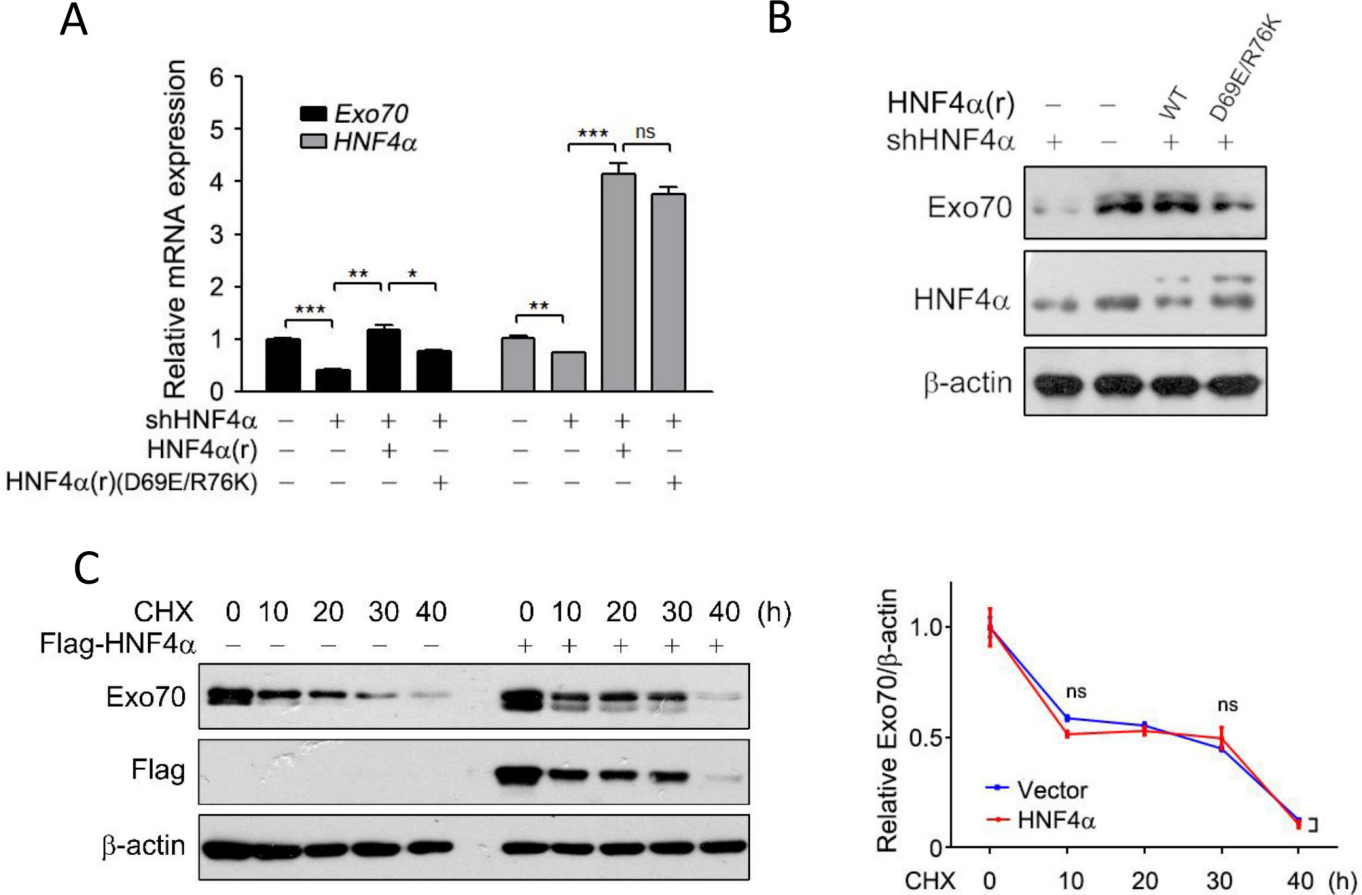
HNF4α transcriptionally regulates the expression of Exo70 (**A**–**B**) The transactivation activity of HNF4α is required to regulate Exo70 expression. Hep G2 cells were transiently transfected with pLL3.7 vector or pLL3.7-shHNF4α plasmid. Twenty-four hours after transfection, cells were transfected with pCMV10 vector, HNF4α silence mutation Flag-HNF4α(r) or Flag-HNF4α(r)(D69E/R76K) plasmid. Another 24 h later, cells were harvested and then subjected to real time PCR (A) and Western blot (B). GAPDH was used as normalization control for real time PCR. The basal level in the vector group were normalized to 1. Data were presented as the means ± s.e.m. from three independent experiments. Differences between two groups as indicated in the graph were assessed by a two tailed unpaired Student's *t*-test. *p < 0.05, **p < 0.01, ***p < 0.001 versus control. (**C**) HNF4α did not alter the protein stability of Exo70. Hep G2 cells were transfected with pCMV10 vector or Flag-HNF4α plasmid. Eight hours after transfection, cells were treated with 10 μM cycloheximide (CHX) for different durations. Cells were then harvested and subjected to Western blot. Expression of Exo70 and β-actin (used as loading control) were detected and quantified by densitometry. Bar graph (right panel) showed the ratios of Exo70 to β-actin, and the basal levels in the groups without CHX treatment were normalized to 1. Data were presented as the means ± s.e.m. from three independent experiments. Differences between two groups as indicated in the graph were assessed by a two tailed unpaired Student's *t*-test, ns nonsignificant versus control.

We then determined whether this regulation is correlated with the transactivation activity of HNF4α. A dominant-negative mutant HNF4α(D69E/R76K), which lacks the ability to recognize the promoter of its target genes [24], were introduced into the Hep G2 cells with endogenous HNF4α knocked down beforehand. Results showed that both mRNA and protein levels of Exo70 were downregulated when HNF4α expression was impaired by shHNF4α constructs; however, reintroduction of HNF4α, but not its dominant-negative mutant HNF4α(D69E/R76K), rescued the mRNA and protein expression levels of Exo70 (Figure 2A–2B). These results thus revealed that HNF4α transcriptionally activated Exo70, and the transactivation activity of HNF4α was critical for its regulation on Exo70 expression.

Non-genomic functions of nuclear receptors or transcription factors were reported in recent years, including modulating post-translational modification and consequently affecting protein stability [25–27]. As such, the effect of HNF4α on the protein stability of Exo70 was also determined. The results showed that exo70 protein levels were decreased in a time-dependent manner when Hep G2 cells were treated with the protein synthesis inhibitor cycloheximide (CHX) (Figure 2C); however, despite leading to higher levels of Exo70 at each indicated time, overexpression of HNF4α didn't change the degradation rate of exo70 under CHX treatment (Figure 2C), indicating that HNF4α didn't affect the protein stability of exo70.

### Identification of Exo70 as a novel transcriptional target of HNF4α

**Figure 3:**
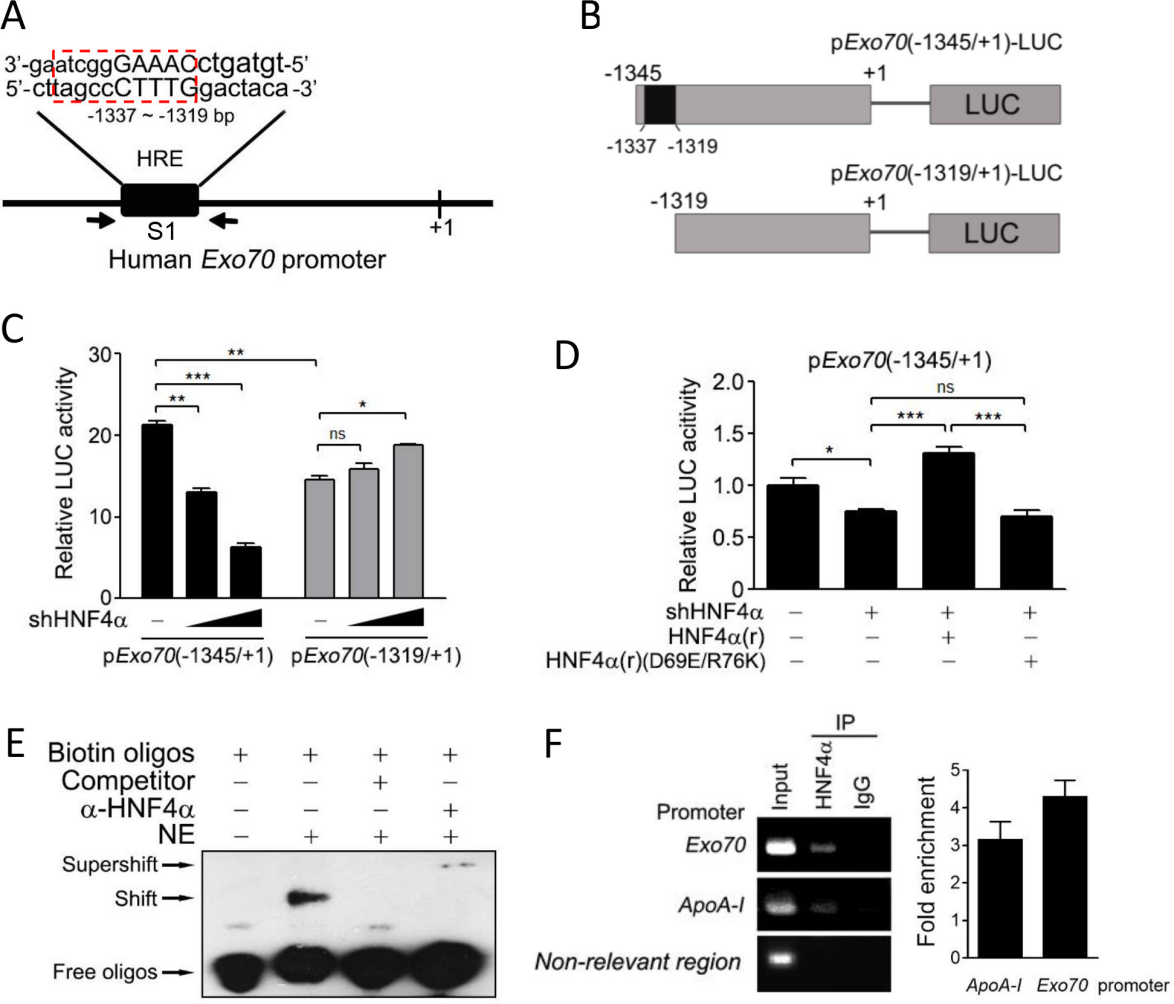
Exo70 is a novel target gene of HNF4α (**A**) Potential HNF4α binding site (HRE) in the promoter of the human *Exo*70 gene. (**B**) A schematic diagram for the different fragments of Exo70 promoter constructed into the luciferase reporter plasmid. (**C**) Effects of HNF4α on the activity of Exo70 promoter. Hep G2 cells were co-transfected with p*Exo*70(−1345/+1)-LUC or p*Exo*70(−1319/+1)-LUC, pLL3.7 or pLL3.7-shHNF4α, and the pRL-TK vector. Forty-eight hours after transfection, cells were harvested and the luciferase reporter assay was carried out. (**D**) The transactivation activity of HNF4α is required to regulate the transcription level of Exo70 promoter. Hep G2 cells were transiently transfected with pLL3.7 vector or pLL3.7-shHNF4α plasmid. Twenty-four hours after transfection, cells were transfected with pCMV10 vector, HNF4α silence mutation Flag-HNF4α(r) or Flag-HNF4α(r)(D69E/R76K), together with p*Exo70*(−1345/+1)-LUC plasmid. After another 36 h, cells were harvested and the luciferase reporter assay was carried out. The basal level of transcriptional activity was normalized to 1. Data were presented as the means ± s.e.m. from three independent experiments. Differences between two groups as indicated in the graph were assessed by a two tailed unpaired Student's *t*-test, *p < 0.05, **p < 0.01, ***p < 0.001, ns nonsignificant versus control. (**E**–**F**) Binding of HNF4α to the human Exo70 promoter was detected by EMSA (E) and ChIP (F). For EMSA, nuclear extract (NE) of Hep G2 cells was incubated with biotin-labeled oligonucleotides containing HRE elements in the *Exo70* promoter. For ChIP, Hep G2 cells were harvested and then the ChIP assay was carried out to detect the interaction between endogenous HNF4α and the *Exo70* promoter using anti-HNF4α antibody. The non-relevant region was PCR with a negative control primer pair which bound about 3 kilobases away from the putative HNF4a binding sites. Fold enrichment was calculated from a ratios between the signals of the *Exo70* promoter (or *ApoA-I* promoter) and the negative control.

The above results suggest that Exo70 may be a novel target gene of the transcription factor HNF4α. A consensus HNF4α binding site described by Sladek [14] was predicted to be located between −1337 and −1319 bp (named S1, Figure 3A) within the human *Exo70* promoter using BIOBASE biological databases online analysis. To further determine the effect of HNF4α on the transcriptional activity of *Exo70* gene promoter and the precise sequence needed for this action of HNF4α, different fragments of Exo70 promoter, the −1345 ∼ +1 region containing S1 and the −1319 ∼ +1 region excluding S1, were constructed into the luciferase reporter plasmid (Figure 3B). As shown in Figure 3C, knocking down HNF4α expression significantly attenuated the transcriptional levels of the −1345 ∼ +1 region within the Exo70 gene promoter, but not that of the −1319 ∼ +1 region (Figure 3C), suggesting that S1 is essential for HNF4α to transactivate *Exo70* gene promoter. Notably, a lower basal transcriptional activity was observed in the −1319 ∼ +1 region as compared to the −1345 ∼ +1 region, providing supplementary evidence that some response element exists in the S1 (Figure 3C). Rescue assay was also carried out. The reintroduction of HNF4α, but not its dominant-negative mutant HNF4α(D69E/R76K), succeeded in regaining the transcriptional activity of the Exo70 gene promoter, which was impaired in advance by knocking down endogenous HNF4α (Figure 3D). This is consistent with the above observation that the transactivation activity of HNF4α is necessary for its modulation on Exo70 expression.

Direct binding of HNF4α to the *Exo70* gene promoter was further confirmed by electromobility shift assays (EMSA) and chromatin immunoprecipitation (ChIP) assays. S1 (−1337 ∼ −1319 region) was used as probe in the EMSA experiment. The results showed that biotin-labeled S1 probes formed complex with some nuclear protein (Figure 3E, shift band), and the specificity of this binding was confirmed by the addition of excess unlabeled S1 probes, which effectively competed for binding the nuclear protein (Figure 3E). The presence of HNF4α protein in the S1-nuclear protein complex was verified by adding anti-HNF4α antibody to the reaction mixture, which led to a further gel-retardation of the nuclear protein-DNA complex (Figure 3E, supershift band). The interaction between the HNF4α and the *Exo70* promoter was further verified *in vivo* by ChIP experiments (Figure 3F). Together, these results indicate that Exo70 is a transcriptional target of HNF4α.

### Exo70 acts downstream of HNF4α to regulate cell cycle

**Figure 4:**
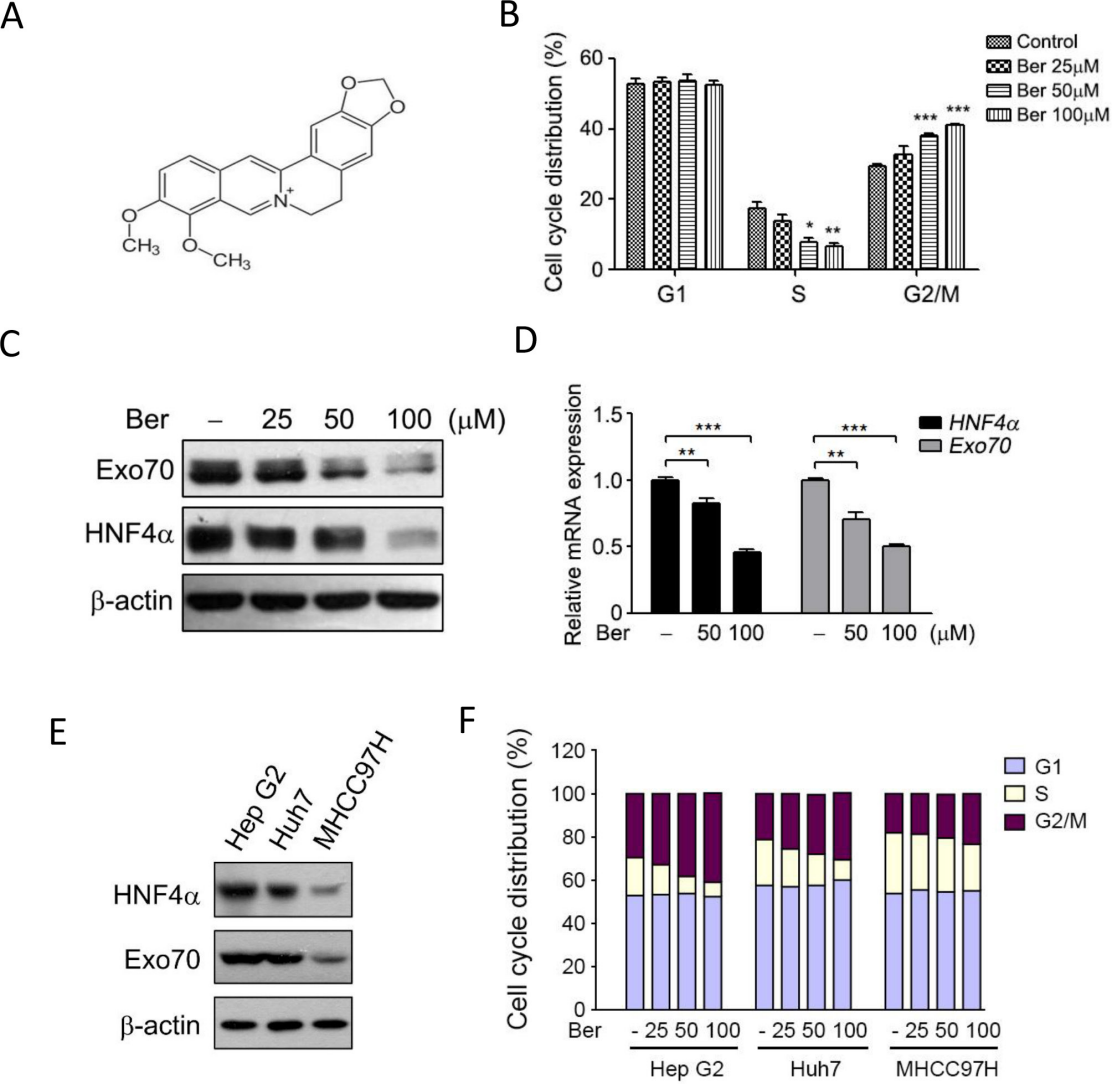
Berberine reduced the expression of HNF4α and Exo70, and induced cell cycle arrest at G2/M phase (**A**) Chemical structure of berberine. (**B**) Berberine arrest the cell cycle of Hep G2 at G2/M phase. Cells were treated with different doses of berberine as indicated for 24 h, and then subjected to cell cycle analysis. (**C**–**D**) Berberine decreased the expression of HNF4α and Exo70. Human hepatoma Hep G2 Cells were treated with berberine (Ber) for 24 h, and then subjected to Western blotting or real-time PCR. The base level in each cell sublines were normalized to 1. Data were presented as the means ± s.e.m. from three independent experiments. Differences between two groups as indicated in the graph were assessed by a two tailed unpaired Student's *t*-test, *p < 0.05, **p < 0.01, ***p < 0.001 versus control. (**E**–**F**) Down-regulation of HNF4α and Exo70 is essential to the G2/M cell cycle arrest in hepatoma cells with berberine treatment. Expression levels of HNF4α and Exo70 in three hepatoma cell lines were analyzed by Western blot (E). Hep G2, Huh7 and MHCC97H were treated with different doses of berberine for 24 h, then subjected to cell cycle analysis.

We and others found that berberine (Figure 4A), a traditional Chinese medicine with anti-tumor effects, could induce G2/M phase cell cycle arrest in hepatoma cells [28] (Figure 4B). However, the related mechanisms were not fully understood. We accidently found that the expression of HNF4α and Exo70 declined with similar trend when cells were treated with different doses of berberine (Figure 4C–4D). To test whether down-regulation of HNF4α and Exo70 is essential to the G2/M cell cycle arrest in hepatoma cells with berberine treatment, several hepatoma cell lines with different expression levels of HNF4α and Exo70 (Figure 4E) were used. Results showed that berberine induced G2/M phase arrest in all these cell lines; however, MHCC97H cell with low HNF4α and Exo70 expression was less affected by berberine than Hep G2 and Huh7 cells with high HNF4α and Exo70 expression (Figure 4F). These results provided hints that HNF4α and Exo70 may be involved in the regulation of cell cycle in hepatic cancer cells.

**Figure 5:**
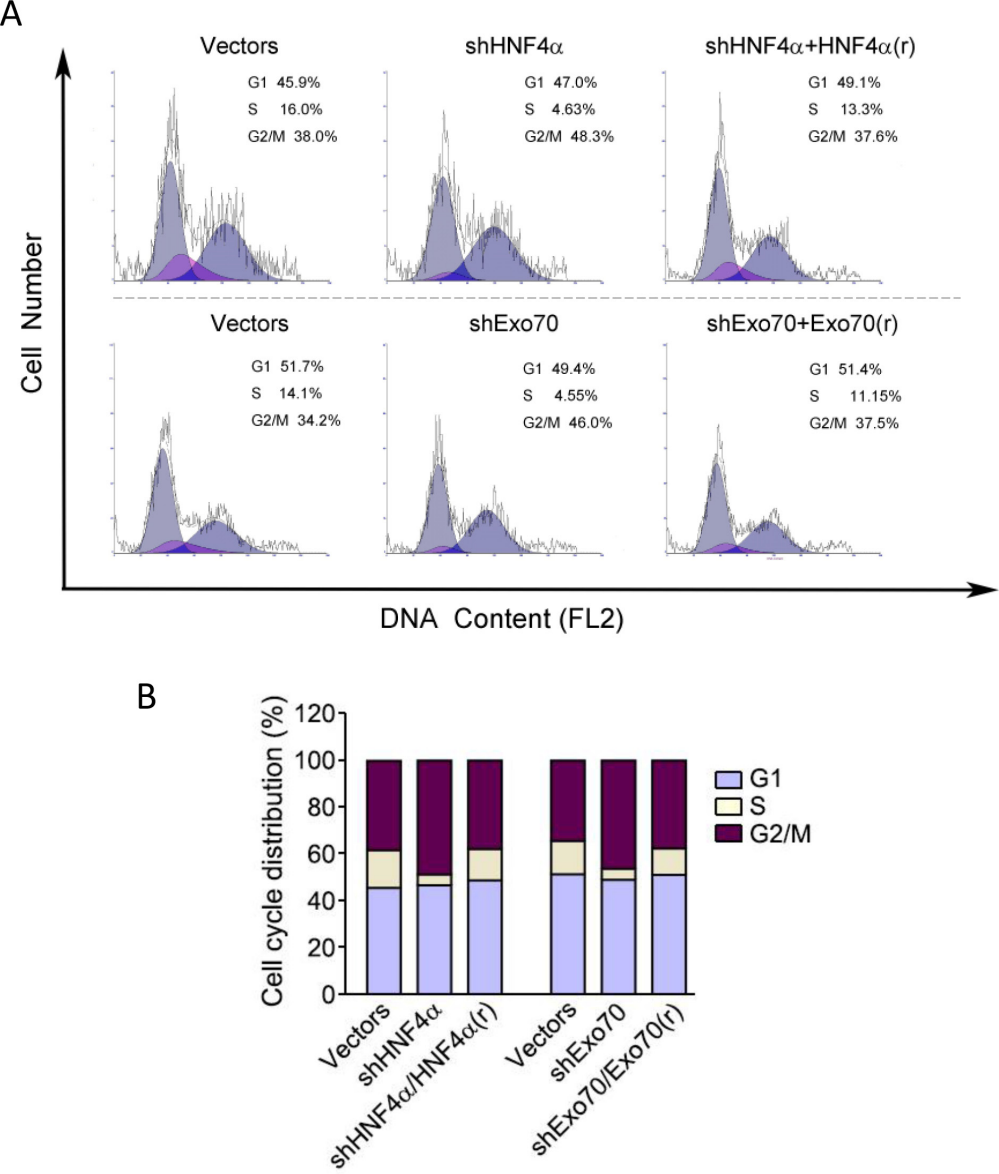
Knocking down either HNF4α or Exo70 can arrest cell cycle of liver cancer cells at G2/M phases A-B. PLC/PRF/5 cells were transiently transfected with pLL3.7-shHNF4α or pLL3.7 vector, or pGV248-shExo70 or pGV248 vector as needed; 24 hours after transfection, cells were transfected with pCMV10 vector, HNF4α silence mutation Flag-HNF4α(r) or Flag-Exo70(rattus) plasmid as needed. Another 24 h later, cells were harvested and then subjected to cell cycle analysis.

To explore the potential roles of HNF4α and Exo70 in cell cycle progression, endogenous HNF4α and Exo70 were knocked down separately and the cell cycle were analyzed. Results showed that knocking down either HNF4α or Exo70 arrested human hepatoma cell cycle at G2/M phase (Figure 5A–5B). Reintroducting HNF4α corrected the dysregulated cell cycle induced by knocking down endogenous HNF4α; similarly, reintroducing Exo70 also reversed the cell cycle progression changed by knocking down Exo70 (Figure 5A–5B), thus confirming the critical roles of HNF4α and Exo70 in the regulation of cell cycle in hepatic cancer cells.

**Figure 6:**
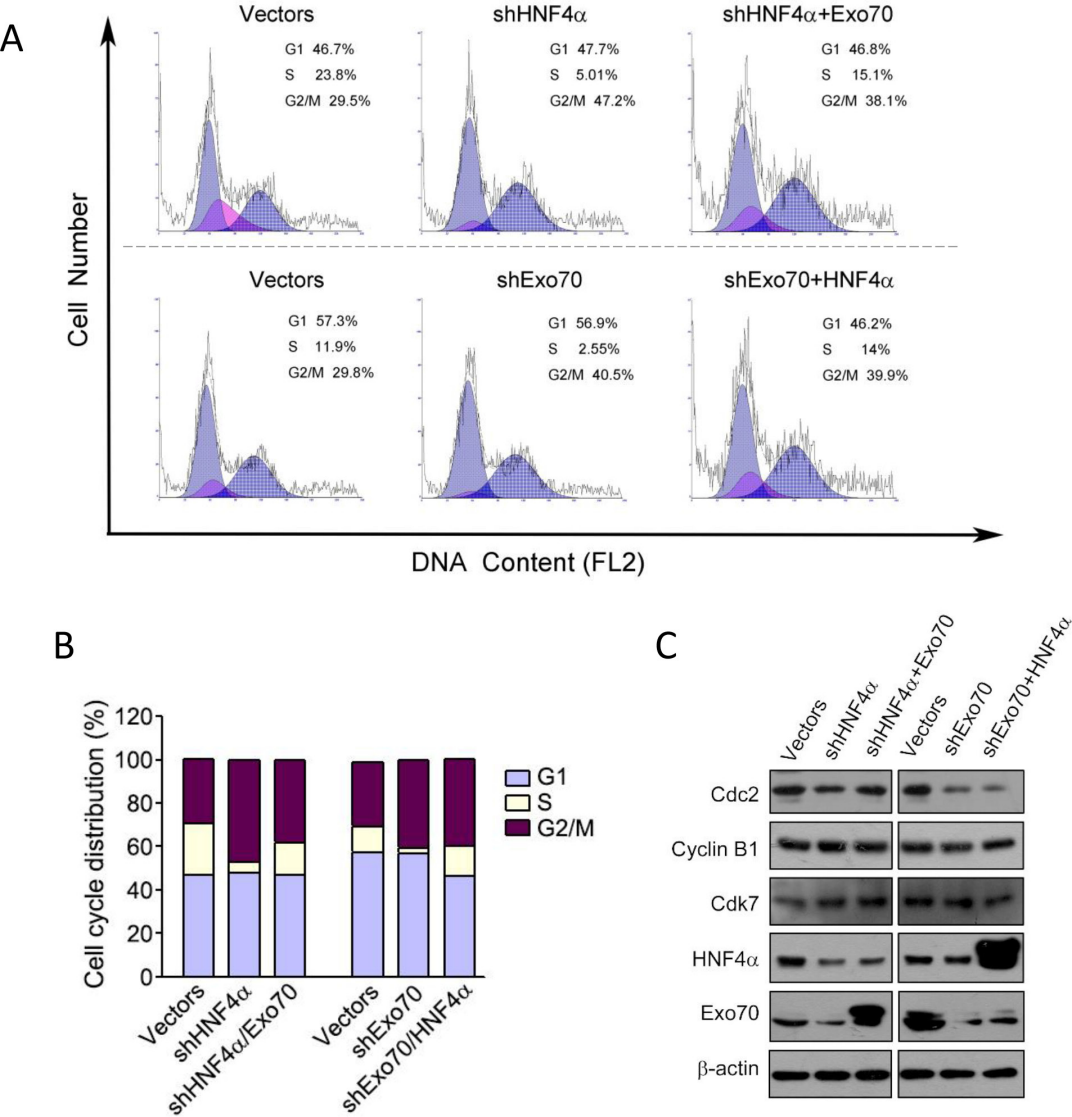
Exo70 acts downstream of HNF4α to regulate cell cycle via Cdc2 PLC/PRF/5 cells were transiently transfected with pLL3.7-shHNF4α or pLL3.7 vector, or pGV248-shExo70 or pGV248 vector as needed. 24 hours after transfection, cells were transfected with pCMV10 vector, Flag-HNF4α or Flag-Exo70(rattus) plasmid as needed. Another 36 h later, cells were harvested and subjected to cell cycle analysis (A-B) or Western blot (C).

As we observe a regulation of Exo70 by HNF4α, we sought to explore whether HNF4α and Exo70 acted in the same axis to regulate hepatoma cell cycle. Overexpression of Exo70 in human hepatoma PLC/PRF/5 cells corrected the dysregulated cell cycle caused by knocking down endogenous HNF4α (Figure 6A–6B); in contrast, overexpressing HNF4α showed no influence on the G2/M cell cycle arrest induced by knocking down endogenous Exo70 (Figure 6A–6B). These results suggested that Exo70 acts downstream of HNF4α to regulate cell cycle progression.

To understand the mechanism of G2/M cell cycle arrest by Exo70 or HNF4α, we examined the expression of several proteins related to cell cycle regulation, including Cdc2, Cyclin B1 and Cdk7. The results showed that knocking down either HNF4α or Exo70 in human hepatoma cells reduced the expression of Cdc2, while had no influence on the expression of Cyclin B1 and Cdk7. In addition, overexpressing Exo70 elevated the level of Cdc2 reduced by knocking down endogenous HNF4α; however, overexpressing HNF4α did not affect Cdc2 expression decreased by knocking down endogenous Exo70. Together, these results indicated that inhibiting HNF4α-Exo70 axis arrested G2/M cell cycle progression partially via suppressing the expression of Cdc2.

## DISCUSSION

Exo70, an important component of the exocyst complex, was reported to promote cell exocytosis, migration, invasion and autophagy [1, 3, 9–11]. However, the regulation of Exo70 is still unknown except that extracellular signal-regulated kinase (ERK) phosphorylation of Exo70 at Ser250 facilitates its interaction with other exocyst subunits and the assembly of the exocyst complex [9]. In this study, Exo70 was found to be transcriptionally regulated by HNF4α in hepatoma cells. Knocking down HNF4α decreased Exo70 protein and mRNA expression (Figure 1) but not its protein stability (Figure 2C), and the transactivation activity of HNF4α was required for its effect on Exo70 expression (Figure 2A–2B). Furthermore, through a series of experiments, we proved that Exo70 is a novel transcriptional target gene of HNF4α (Figure 3).

Exo70 was reported to stimulate tumor invasion and migration as knocking down endogenous Exo70 in breast cancer cells greatly suppressing their invasion and migration ability [9, 10]. However, the invasion and migration ability of human cervical carcinoma (HeLa) and hepatoma (Hep G2) cells were not affected by knocking down Exo70 (data not shown). Therefore, it's likely that Exo70 was not a critical protein involved in the regulation of hepatoma cell migration and invasion. Nevertheless, a novel function of hepatic Exo70 in the regulation of cell cycle progression in hepatoma cells was identified in this study. We found that reduced expression of Exo70 and HNF4α was important for berberine-induced G2/M cell cycle arrest in hepatoma cells (Figure 4), suggesting their potential roles in cell cycle regulation. We further proved that knocking down either HNF4α or Exo70 in hepatoma cells arrested cell cycle progression at G2/M phase (Figure 5), and Exo70 acted downstream of HNF4α to promote G2/M transition via increasing Cdc2 expression in HCC cells (Figure 6). Actually, it was not the first time that exocyst subunit was reported to participate in cell cycle control. Sec8 and Sec6 were previously found to regulate G1/S transition in oral squamous cell carcinoma cells via p21^Waf1/Cip1^ and p27^Kip1^, respectively [29, 30]. It appears that these subunits can also have their own specific biological functions via distinct molecular mechanisms, in addition to work as members of the exocyst complex.

We have long been making effects to explore the molecular mechanisms underlying the antitumor effect of berberine, a traditional Chinese medicine, especially how it arrests cell cycle. In the present study, we accidently found that berberine reduced the expression of HNF4α and Exo70, which is critical for its effect on G2/M transition of hepatoma cells (Figure 4). And we further demonstrate the role and mechanisms of HNF4α and Exo70 in cell cycle regulation (Figures 5–6). It would also be interesting to investigate the mechanism how berberine can reduce the mRNA expression of HNF4α. Previous studies showed that berberine could bind to the poly(A) tail on retinoblastoma (Rb) mRNA to suppress its degradation [31], suggesting that the binding of berberine to mRNA molecular may modulate the stability of mRNA. We thus conjecture that berberine may promote the degradation of HNF4α mRNA via binding to its poly(A) tail, which will be explored in our further studies.

Recently, conflicting reports have defined HNF4α as both tumor promoter and tumor suppressor in hepatocellular carcinoma (HCC). The expression of HNF4α was significantly increased in HCC as compared to the adjacent non-tumor tissues, indicating a tumor promoting role of HNF4α in HCC [32]; however, another study reported that overexpression of HNF4α decreases the tumorigenicity of hepatoma cells [33], defining HNF4α as a hepatoma tumor suppressor. Our present study found that HNF4α works as a tumor promoter by transactivating exo70, which increases Cdc2 expression leading to G2/M transition. Owing to the pretty high expression of HNF4α in hepatoma cells, knocking down rather than overexpressing HNF4α would be a good choice to investigate the function of HNF4α in HCC, just as we did in the present study.

**Figure 7:**
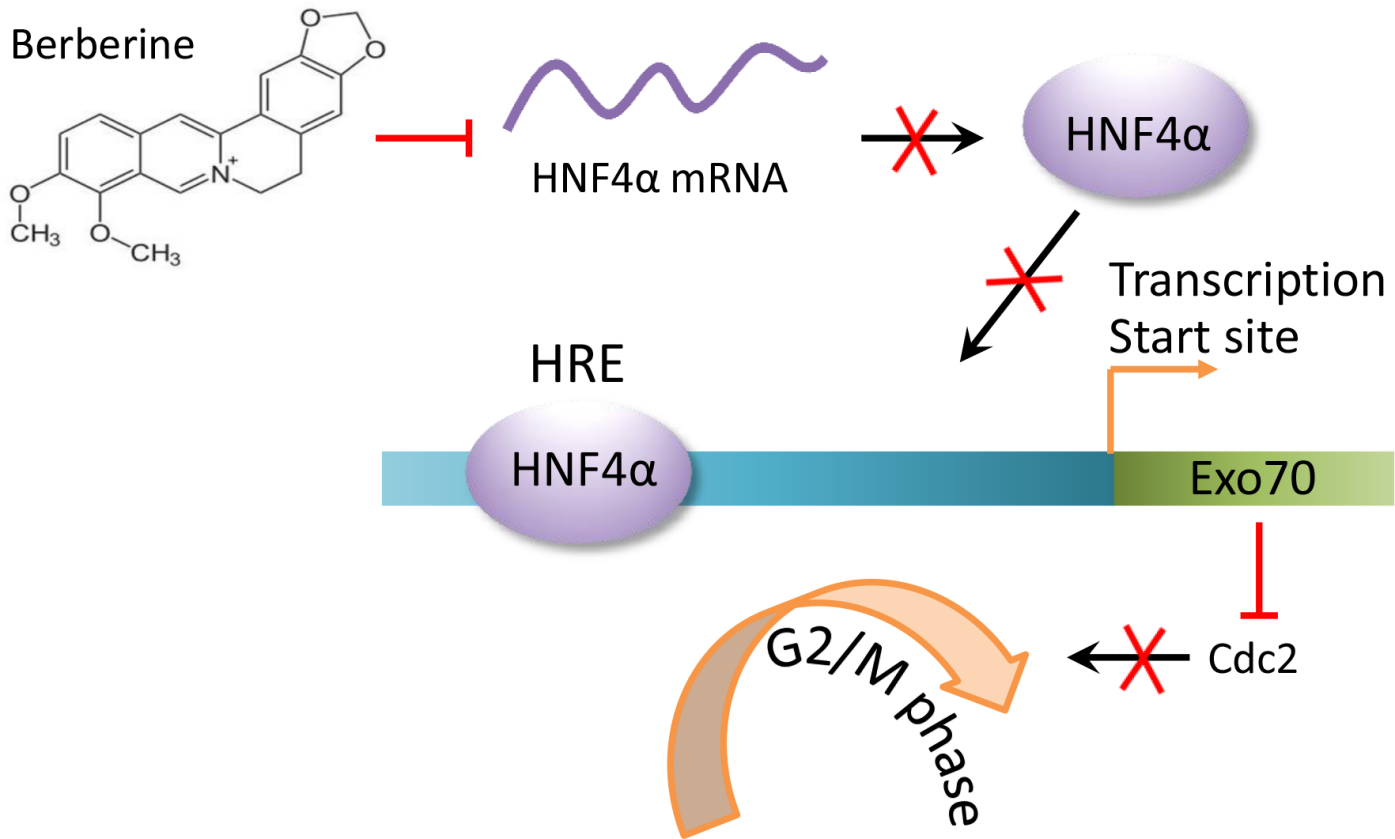
A schematic diagram for cell cycle arrest induced by downregulating HNF4α-Exo70 axis The reduction in HNF4α mRNA levels induced by berberine results in a decreased HNF4α protein expression levels. Less HNF4α protein binding to the HNF4α response element (HRE) in the *EXo70* promoter leads to decreased transcription of Exo70. Subsequently, the reduced Exo70 protein level causes hepatoma cell cycle arrest at G2/M phase.

Together, our results identify Exo70 as a novel transcriptional target of HNF4α and contributes to the regulation of cell cycle progression in hepatoma cells (Figure 7), which may provide a suitable target for the development of antitumor strategies.

## MATERIALS AND METHODS

### Cell culture, transfection and luciferase reporter assays

Human hepatoma cell lines Hep G2 and PLC/PRF/5 were purchased from the Institute of Cell Biology, China. Human hepatoma cell lines Huh7 and MHCC97H, human bile duct cancer cell line QBC939 were purchased from ATCC, USA. Hep G2, Huh7 and MHCC97H were cultured in Dulbecco's modified Eagle's medium (Gibco, Grand Island, NY, USA), PLC/PRF/5 and QBC939 cells were cultured in modified Eagle's medium (Gibco) and RPMI-1640 (Gibco), respectively. All the medium was supplemented with 10% fetal bovine serum (HyClone, Logan, UT, USA), 100 U of penicillin, and 100 μg/ml of streptomycin (Life Technologies, Carlsbad, CA, USA).

Cell transfections were carried out with the Lipofectamine 2000 transfection reagent (Invitrogen) according to the manufacturer's instructions. Luciferase assays were performed using the Luciferase Reporter Assay Kit (Promega, Madison, WI, USA), and luminescence was measured on a GloMax 20/20 Luminometer (Promega) in accordance with the manufacturer's guidelines. Firefly luciferase activity normalized to Renilla luciferase activity represents the luciferase activity.

### Reagents and antibodies

Cycloheximide (CHX, cat. #C7698), propidium Iodide (PI, cat. #P4170) and deoxyribonucleic acid sodium salt from salmon testes (cat. #D1626) were purchased from Sigma Aldrich, St. Louis, MO.

Mouse anti-β-actin antibody (cat. #ab3280) was purchased from Abcam. Goat anti-HNF4α (cat. #sc-6556) and rabbit anti-Exo70 (cat. #sc-135082) antibodies, normal goat IgG (cat. #sc-2028), and protein A/G plus-agarose (cat. #sc-2003) were purchased from Santa Cruz Biotechnology, Santa Cruz, CA, USA. Rabbit anti-Cyclin B1 (cat. #4138), mouse anti-Cdk7 (cat. #2916) and anti-Cdc2 (cat. #9116) antibodies were purchased from Cell Signaling Technology, Beverly, MA, USA. Mouse anti-Flag (cat. #F3165) and mouse anti-HA (cat. #H9658) antibodies were purchased from Sigma Aldrich, St. Louis, MO.

### Plasmids

Human HNF4α2 (purchased from Addgene, USA) and rattus Exo70 (kindly provided by Wei Guo, University of Pennsylvania, Philadelphia, USA) were separately subcloned into pCMV10/3×FLAG (Sigma Aldrich, St. Louis, MO). Two different regions of human Exo70 promoter, −1345∼+1 and −1319∼+1, were cloned and inserted into pGL6-TA (Beyotime, Shanghai, China). HNF4α2 silence mutation that could not be recognized by shHNF4α was used for the RNAi rescue experiments (named as HNF4α(r), r means rescue). Primers for HNF4α2 silence mutation were: 5′-CAGAATGAGCGGGACCGGATATCGACACGAAG GTCAAGCTATGAGGAC-3′ (for-ward), 5′-GTCCTCAT AGCTTGACCTTCGTGTCGATATCCGGTCCCGCTCA TTCTG-3′ (reverse). The point mutations of HNF4α2 were constructed using the QuikChange Mutagenesis Kit (Stratagene, La Jolla, CA, USA) according to the manufacturer's instructions and verified using sequencing (Invitrogen, Guangzhou, China).

The shRNA sequences targeting human HNF4α was 5′-GATCAGCACTCGAAGGTCAA-3′. The shRNA sequences targeting human Exo70 was 5′-TGCA GGAGAATGTTGAGAA-3′. The shRNA control (scramble) sequence was: 5′-GGCTACGTCCAGGAGCGCACC-3′. Oligonucleotides (Invitrogen, Guangzhou, China) were annealed and inserted into the pLL3.7 vector (for sh HNF4α) or pGV248 (for shExo70).

### Cell extraction and western blot analyses

Cells were lysed in ice-cold RIPA buffer (50 mM Tris (pH 7.4), 150 mM NaCl, 1% NP-40, 0.5% sodium deoxycholate, and 0.1% SDS) containing 1 mM PMSF (Sigma Aldrich, St. Louis, MO) and protease inhibitors (Roche, Indianapolis, IN, USA). The protein concentration of cell lysates was measured by BCA kit (Thermo Scientific, Rockford, IL, USA), and then subjected to SDS-PAGE. Proteins were transferred to nitrocellulose membrane and incubated with antibodies. Immunoreactive bands were detected using Enhanced Chemiluminescence (ECL) system (Bio-Rad, Hercules, CA, USA).

### Real time PCR

Total RNA was extracted using Trizol reagent (TaKaRa, Dalian, China) and then reverse-transcribed to cDNA using Primescript^™^ RT reagent kit (TaKaRa, Dalian, China). Real-Time PCR was then carried out using the SYBR Green I fluorescent dye (SYBR^®^ Premix Ex Taq^™^ II, TaKaRa, Dalian, China) and the StepOnePlus^™^ real-time PCR system (Applied Biosystems, Australia). The thermal cycling consisted of an initial pre-degeneration at 95°C for 30s, followed by 40 cycles of de-naturation at 95°C for 5s and annealing/extension at 60°C for 30s. Primers used were listed as follows:

*HNF4α*, 5′-TGCGACTCTCCAAAACCCTC-3′ (forward)

5′-ATTGCCCATCGTCAACACCT-3′ (reverse)

*Exo70*, 5′-GGAGTATTTCCAGGACAACAGC-3′ (forward)

5′-AAGATGAGCACGGGCGAGA-3′ (reverse)

*GAPDH*, 5′-TGCACCACCAACTGCTTAGC-3′ (forward)

5′-GGCATGGACTGTGGTCATGAG-3′ (reverse)

### Electrophoretic mobility shift assay (EMSA)

Nuclear extract were prepared as described previously [34]. Double-stranded biotin-labeled oligonucleotides (Invitrogen, Guangzhou, China) harboring the predicted HNF4α binding site on the *Exo70* promoter (5′-CCTACTTAGCC*CTTTG*GACTACACAAGG-3′) were used as probes. Competitors were non-biotin-labeled oligonucleotides. The EMSA experiment was carried out using LightShift Chemiluminescent EMSA Kit (Thermo Scientific, cat. #20148) in accordance with the manufacturer's guidelines. For supershift, anti-HNF4α antibody (Santa Cruz Biotechnology, Santa Cruz, CA, USA) was added to the nuclear extract after incubation with biotin-labeled probes. When the binding reaction finished, the mixtures were loaded onto a 6% polyacrylamide gel and subjected to electrophoresis, then transferred to a nylon membrane (Hybond-N, Amersham Pharmacia Biotech, Little Chalfont, UK). The biotin-labeled doublestranded DNA was then visualized using the Chemiluminescent Nucleic Acid Detection Module in this Kit.

### Chromatin immunoprecipitation (ChIP) assay

Cells were cross-linked with 0.75% formaldehyde for 10 min at room temperature, and stopped by the addition of glycine to a final concentration of 0.125 M. Cells were collected, resuspended in ChIP sonication buffer [35, 36] and then sonicated. After centrifugation, the supernatant was diluted 10 folds with dilution buffer, and immunoprecipitation was performed with anti-HNF4α antibody (Santa Cruz Biotechnology, Santa Cruz, CA, USA) or goat IgG (Santa Cruz Biotechnology) at 4°C overnight. After that, protein A/G agarose beads (pretreated with deoxyribonucleic acid (DNA) from salmon testes for 30 min at room temperature) were added, and incubated for another 1 hour at 4°C. Beads were then washed twice in low salt buffer, twice in high salt buffer, twice in LiCl buffer and once in TE buffer. The protein-DNA complexes were then extracted with 200 elution buffer (containing 0.5 mg/mL RNaseA), and their crosslinks were reversed by heating at 65°C for 5 h. DNA was extracted with DNA Purification Kit (TianGen, cat. #DP130227). PCR was performed with 10 ng template DNA and the primers used were listed as follows:

Human *Exo70* promoter, 5′-CAAGACTCCGT CTCAAGAA-3′ (forward)

5′-CTCAGGCAAGAGGACAAT-3′ (reverse)

Human *ApoA-I* promoter, 5′-GCTTGCTGTTTGC CCACT-3′ (forward)

5′-GGTCCTGGCAATGTGGAA-3′ (reverse)

Non-relevant region, 5′-TAGCCAACCTG GTGAAGC-3′ (forward)

5′-TGTACGTGACCACCTTTGT-3′ (reverse)

### Cell cycle analysis

Cells were trypsinized (0.05% trypsin without EDTA), collected and fixed with 70% ethanol at 4°C overnight and then treated with 1.0 mg/ml RNase A (Roche) for 10 min to digest RNA. After that, cells were washed twice with PBS and stained with 50 mg/ml of propidium iodide (Sigma) for 15 min. Samples were then analyzed by a flow cytometer (Cyflow Space, Partec, Germany) and the ModFit LT Tutorial Series software.

### Statistical analysis

Results were presented as means ± SEM. Statistical significance was determined by Student's *t*-test using GraphPad Prism 5 (Graphpad Software). Differences were considered statistically significant at the *p* < 0.05 level.
